# Quantifying the properties of selective laser sintered nylon foot orthoses under linear loading

**DOI:** 10.1186/1757-1146-7-S2-A7

**Published:** 2014-11-14

**Authors:** Jari Pallari, Harriet Slack, Alastair Masson, Greg Jackson, Javier Munguia, Kenny Dalgarno

**Affiliations:** 1 Peacocks Medical Group, Benfield Business Park, Newcastle upon Tyne, UK; 2 School of Mechanical and Systems Engineering, Newcastle University, Newcastle upon Tyne, UK

## Background

Before launching a new bespoke foot orthotic (FO) product, it is necessary to understand how it works under compressive loads. Additive manufacturing and the selective laser sintering process in particular can enable a number of innovative and functional features to be integrated to the FO device. A simple example of this design freedom is to adjust the shell thickness of an FO. This research is an effort to measure the displacement of the arch as the FO is loaded with a certain force and how much different FO shell thicknesses impact the amount of displacement with the same load.

## Methods

The FO’s tested were designed as bespoke FO's for a patient. They were identical apart from their thickness which varied from 1 to 3.5mm in 0.5mm intervals. The test was set up as follows:

• The FO was clamped onto the Instron 4505 test machine at the heel with the aid of the sand bag to spread the clamping load. The FO was also clamped on the 5th metatarsal.

• The instrument head pressing on the part was placed on the estimated position of the navicular bone.

• The testing start point varied between tests as it was determined by eye. This means that the displacement of the machine test head before contact with the orthosis will vary slightly between tests.

• A maximum load of 400N was applied at the rate of 100mm/min.

Each orthosis compressed 3 successive times in order to determine any changes in behaviour. This process was repeated for all six orthoses. The same experiment was the set up again to simulate the test being repeated in a different laboratory.

## Results

The line versus displacement curves for the orthoses generally follow the same kind of pattern in terms of their behaviour and the variation between subsequent tests. The load required to flatten the orthosis (approximately 20mm) can give an indication of the flexibility. The thinnest orthosis (and hence most flexible) took approximately 16N to reach the ground whereas the thickest (and hence most rigid) book approximately 395N to reach the ground. Generally as an orthosis is subjected to more testing, the load required to produce a certain displacement decreased. The results will be presented in full in the presentation.

## Conclusions

As expected, as the thickness of the orthosis increased, the load applied to cause the same displacement also increased. The pattern of the load vs displacement changed from a complex pattern in load to a more linear and predictable increase as the thickness of the FO increased. This work can be seen as the initial step in engineering a foot orthotic and in quantifying its properties. Future work would include creating a finite element model that can be verified with physical measurements and using this model to optimise the response of the FO in certain loading conditions, or to reduce the mass and size of the FO.

